# *In Silico* Studies on Psilocybin Drug Derivatives Against SARS-CoV-2 and Cytokine Storm of Human Interleukin-6 Receptor

**DOI:** 10.3389/fimmu.2021.794780

**Published:** 2022-01-14

**Authors:** Faez Iqbal Khan, Fakhrul Hassan, Dakun Lai

**Affiliations:** ^1^ School of Electronic Science and Engineering, University of Electronic Science and Technology of China, Chengdu, China; ^2^ Faculty of Rehabilitation and Allied Health Sciences, Riphah International University, Islamabad, Pakistan

**Keywords:** psilocybin, SARS-CoV-2, Mprotease, COVID-19, metabolite, mushroom

## Abstract

Various metabolites identified with therapeutic mushrooms have been found from different sources and are known to have antibacterial, antiviral, and anticancer properties. Over thousands soil growth-based mushroom metabolites have been discovered, and utilized worldwide to combat malignancy. In this study, psilocybin-mushroom that contains the psychedelic compounds such as psilacetin, psilocin, and psilocybine were screened and found to be inhibitors of SARS-CoV-2 Mprotease. It has been found that psilacetin, psilocin, and psilocybine bind to Mprotease with −6.0, −5.4, and −5.8 kcal/mol, respectively. Additionally, the psilacetin was found to inhibit human interleukin-6 receptors to reduce cytokine storm. The binding of psilacetin to Mprotease of SARS-CoV-2 and human interleukin-6 receptors changes the structural dynamics and Gibbs free energy patterns of proteins. These results suggested that psilocybin-mushroom could be utilized as viable potential chemotherapeutic agents for SARS-CoV-2.

## Introduction

Coronaviruses (CoVs) represent the family Coronaviridae and sub-family Orthocoronavirinae. The sub family Orthocoronavirinae is further divided into four genera namely, α-coronavirus, β-coronavirus, γ-coronavirus, and δ coronavirus. The host of α- and β-coronaviruses are mammals, while the host of γ- and δ-coronaviruses are birds (1, 2). Some of the β-coronavirus like Severe Acute Respiratory Syndrome CoV 1 & 2 (SARS-CoV1 and SARS-CoV2), and Middle-East Respiratory Syndrome CoV (MERS-CoV) are the leading cause of pneumonia in this century. The outbreaks of SARS-CoV1, MERS-CoV and SARS-CoV2 were caused by zoonotic viruses in 2003, 2012 and 2019-20 with fatality ratio of 10%, 35% and 5%, respectively (3–8). The International Virus Classification Commission (ICTV), termed this 2019 novel CoV as SARS-CoV-2 (9, 10).

SARS-CoV-2 possesses the positive-sense single‐stranded ~30 kb RNA genome and consists of 12 open reading frames. The genes located on the 3′-terminus of the virus genome code for four structural proteins such as membrane glycoprotein (M), envelope (E), nucleocapsid (N), spike (S) and eight accessory such as 3a, 3b, p6, 7a, 7b, 8b, 9b, and ORF14 proteins (11, 12). ORFIa and ORF1ab genes encode the non-structural proteins that form the replication/transcription complex, respectively. These genes such as ORFIa and ORF1ab encode two polypeptides called pp1a and pp1ab, respectively. The polypeptides are directly translated and catalytically processed by main protease (Mprotease).

Mprotease is accountable for the cleavage of polyprotein downstream of non-structural protein 4 to form distinctive non-structural proteins that show important roles in the life cycle of the virus (13, 14).

The phenomenal spread of the current COVID-19 pandemic to every part of the sphere has lifted the key concerns for the healthcare system. To combat this pandemic, the researchers are using all possible approaches and practices to inhibit the synthesis of crucial non-structural viral proteins, inhibition of the viral replicase enzyme, inhibition of formation of viral-RNA, prevention of self-assembly of viruses or to boost the human immune response against the virus (15, 16). Main protease Mprotease or 3CLpro has a significant part in SARS-CoV-2 maturation and replication. Therefore, Mprotease is a striking and potential target for the anti-coronavirus drug development to tackle this current COVID-19 pandemic (17, 18).

In several studies, psilocybin drugs have been tested against COVID-19 for mental health problems caused by the pandemic, and the results of the clinical trials indicate that psychedelics are physiologically and psychologically safe (19). Psilocybin has been reported to be effective in treating depression recently (20). A study showed that psilocybin therapy has the potential to play an important therapeutic role in treating a variety of psychological disorders after COVID-19 (21). The COVID-19 poses challenges to mental health, but it also offers the opportunity to develop promising new therapeutic approaches.

Recently, researchers have designed a peptide inhibitor against the receptor-binding domain (RBD) of the SARS-CoV-2 spike protein. It was found to bind at the RBD of the SARS-CoV-2 spike at the Angiotensin-converting enzyme 2 (ACE2) binding site with high affinity (22). It has been suggested that the blockage of SARS-CoV-2 spike protein binding to ACE2 in the presence of this peptide may prevent virus entry into host cells (23).

Here, we applied molecular docking and molecular dynamics simulations to study the interaction of different metabolites of psilocybin mushrooms such as psilocybine, psilocin, and psilacetin with Mprotease of SARS-CoV-2. Our findings revealed that psilacetin might inhibit the Mprotease of SARS-CoV-2 and reduce the cytokine storm by decreasing the expression of human interleukin-6 receptors (HIK6). Several clinical trials are underway regarding the effectiveness of these mushroom compounds.

## Materials and Methods

### Drug Assessment

The compounds such as psilacetin, psilocin, and psilocybine were evaluated by Lipinski filter to check drug-likeness (24). *In silico* pharmacokinetic parameters were estimated to analyse the absorption, distribution, metabolism, and excretion (ADME) properties and toxicity of psilacetin, psilocin, and psilocybine. The ADME, carcinogenicity and toxicity were predicted by SwissADME (25), CarcinoPred-EL (26), and PreADMET servers, respectively.

### Docking Studies

The molecular interactions of psilacetin, psilocin, and psilocybine with SARS-CoV-2 Mprotease (PDB: 6LU7) were achieved by means of AutoDock and AutoDock vina (27). The ligands were docked by defining a grid box with spacing of 1 Å and size of 12 × 22 × 14 pointing in x, y and z directions around the protein active site of coordinates −9.9454 × 11.9130 × 68.3144, respectively. The grid size and coordinates are limited to only important active residues of interest. The Lamarckian Genetic Algorithm was used as the search algorithm with default parameter values. The best-docked postures of Mprotease-psilacetin, Mprotease-psilocin, and Mprotease-psilocybine were chosen based on binding energy and proper orientations of ligands in the active pocket of protein. PyMol (28) and Discovery Studio visualizer (BIOVIA) were used for the visualization of the docked complex. The detailed methodology has been reported previously (29, 30).

### MD Simulations

MD simulation is executed effectively to understand structure-function relationships in a protein (31–33). MD simulations were executed on Mprotease, Mprotease-psilacetin, HIK6, and HIK6-psilacetin at 298 K at the molecular mechanics level implemented in GROMACS 2018.2 (34) using the GROMOS96 43a1 force-field. PRODRG server generated the topology files of psilacetin, psilocin, and psilocybine molecules. The Mprotease, Mprotease-psilacetin, HIK6, and HIK6-psilacetin were soaked in a cubic box of water molecules using the *gmx editconf* module for creating boundary conditions. The *gmx solvate* module was used for the solvation.

The spc216 water model was used to solvate the system. The charges on the Mprotease, Mprotease-psilacetin, HIK6, and HIK6-psilacetin were neutralized by addition of Na^+^ and Cl^-^ ions using *gmx genion* module to preserve physiological concentration (0.15 M). A total number of 84/81, 84/82, 195/198, and 195/199 Na^+^/Cl^-^ were added in case of Mprotease, Mprotease-psilacetin, HIK6, and HIK6-psilacetin, respectively. The system was further minimized using 1500 steps of steepest descent.

Equilibration took place in two phases using NVT and NPT ensembles. The particle-mesh Ewald method was applied after the equilibration phase (35) was applied. The production phases consisted of 100 ns and were conducted at a temperature of 298 K. The detailed methodology has been published in various publications (36–40).

### Principal Component Analysis

PCA or ED was performed for Mprotease, Mprotease-psilacetin, HIK6, and HIK6-psilacetin by diagonalizing the covariance matrix C:


(1) $$C_{ij}=<\left(r_{i}-〈r_{i}〉\right)\times \left(r_{j}-〈r_{j}〉\right)\left(i,j=\;1,2,3,\ldots ,3N\right)$$

The *r_i_* denotes the Cartesian coordinate, i^th^ Cα atom, *N* signifies the number of Cα atoms, and the <*r_i_* > indicates time average over all the configurations.

### Gibbs Free Energy Landscape

GFE landscape can suggest structural and conformational changes in Mprotease, Mprotease-psilacetin, HIK6, and HIK6-psilacetin molecules. For 2D and 3D conformational depiction, the GFE landscape was projected on PC1 and PC2 for Mprotease, Mprotease-psilacetin, HIK6, and HIK6-psilacetin, respectively.


(2) $$G_{\left(\text{PC}1,\text{ PC}2\right)}=-k_{\text{B}}T1\text{n}P_{\left(\text{PC}1,\text{ PC}2\right)}$$

The *k*_B_, *T* and *P*_(_ *_PC1, PC2_*_)_ denote Boltzmann constant, temperature, and normalized joint probability distribution for Mprotease, Mprotease-psilacetin, HIK6, and HIK6-psilacetin respectively.

## Results and Discussions

### Drug Likeness

Clinical trials of drugs fail for a variety of reasons, including toxicity and poor pharmacokinetic properties. Pharmacokinetic profiles for psilacetin, psilocin, and psilocybine were calculated to determine their bioavailability. Lipinski’s rule of five assists in distinguishing between drug-like and non-drug-like molecules. A good drug should have these parameters: mass (< 500 Dalton), lipophilicity (LogP < 5), hydrogen bond donors (< 5), hydrogen bond acceptors (< 10) and molar refractivity (40-130). The Lipinski filter and ADMET properties of psilacetin, psilocin, and psilocybine are listed in **Table 1**. These drugs passed Lipinski filters and were non-carcinogenic.

**Table 1:** Lipinski filter and ADMET properties of psilacetin, psilocin, and psilocybine.

Drugs	Lipinski Rule of Five					Carcinogen	Drug likeness	Violations
	Mass	H-donor	H-acceptor	LogP	Molar Refractivity			
Psilacetin	246	1	3	2.2	71.9	No	Qualified	0
Psilocin	204	2	2	2.0	62.2	No	Qualified	0
Psilocybine	284	3	5	1.7	73.2	No	Qualified	0

### Interactions With SARS-CoV-2 Mprotease

**Figure 1:**
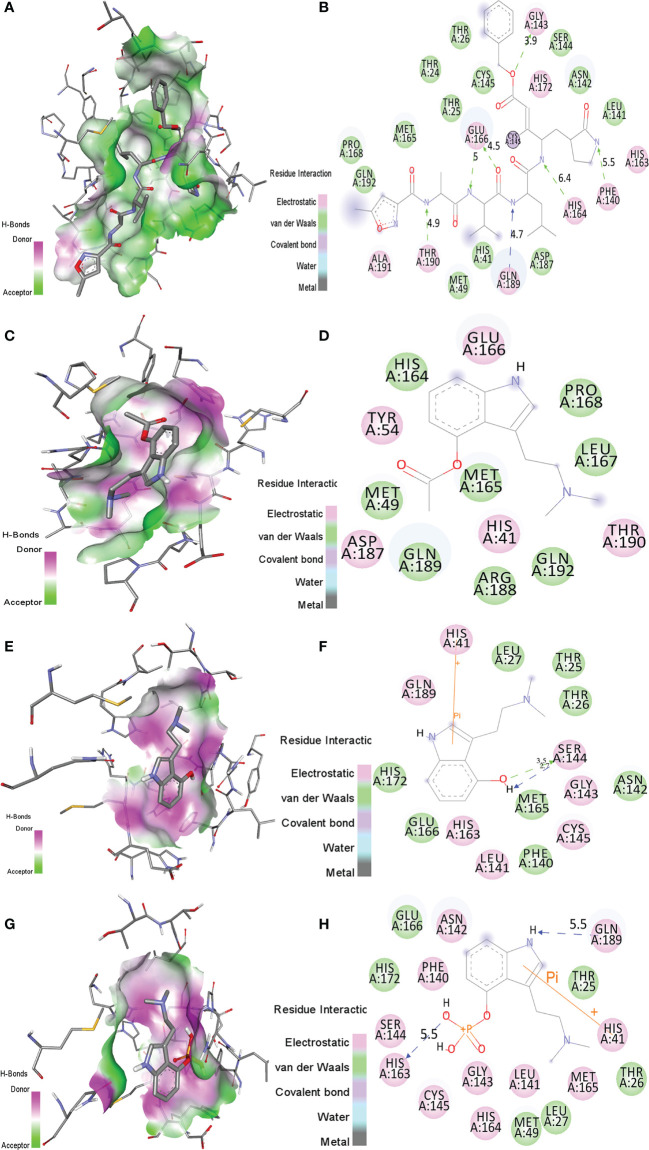
The molecular interactions of **(A, B)** N3 inhibitor, **(C, D)** psilacetin, **(E, F)** psilocin, and **(G, H)** psilocybine with the active pocket of SARS-CoV-2 Mprotease. The structure indicated different residual interactions.

The binding orientations of psilacetin, psilocin, and psilocybine with SARS-CoV-2 Mprotease (PDB: 6LU7) were analyzed by superimposing the inhibitor N3 present in the pocket of Mprotease. The co-crystallized inhibitor N3 showed electrostatic (ES) interactions with residues Phe140, Gly143, His163, His164, His172, Glu166, Gln189, Thr190, and Ala191 of Mprotease. Further, it shows van der Waals (vdW) contacts with residues Thr24, Thr26, Thr25, Met49, His41, Asn142, Leu141, Cys145, Ser144, Pro168, Met165, Asp187, and Gln192 of SARS-CoV-2 Mprotease. The N3 inhibitor was covalently linked with Cys145 of Mprotease (**Figures 1A, B**).

The binding energy of psilacetin with Mprotease was -6.0 kcal/mol. The psilacetin showed ES interactions with residues His41, Tyr54, Glu166, Asp187 and Thr190 of SARS-CoV-2 Mprotease. Further, it showed vdW interactions with residues Met49, His164, Met165, Ley167, Pro168, Arg188, Gln198, Gln192 of SARS-CoV-2 Mprotease (**Figures 1C, D**).

The binding energy of psilocin with Mprotease was found to be -5.4 kcal/mol. The psilocin displayed ES interactions with residues His41, Leu141, Gly143, Ser144, Cys145, His163, and Gln189 of SARS-CoV-2 Mprotease. Further, it shows vdW interactions with Thr25, Thr26, Leu27, Phe140, Asn142, Met165, Glu166, and His172 of SARS-CoV-2 Mprotease (**Figures 1E, F**).

The binding energy of psilocybine with Mprotease was -5.8 kcal/mol. The psilocybine showed ES contacts with His41, Asn142, Phe140, Leu141, Gly143, Cys145, Ser144, His164, His163, Met165, and Gln189 of Mprotease. Further, it shows vdW interactions with residues Thr25, Thr26, Leu27, Met49, Glu166, and His172 of Mprotease (**Figures 1G, H**). The detailed interacting residues are mentioned in **Table 2**. It has been found that psilacetin strongly binds with Mprotease. The binding of these compounds was further analyzed using MD simulations.

**Table 2:** The ES, vdW and covalent interactions of inhibitor N3, psilacetin, psilocin, and psilocybine with SARS-CoV-2 Mprotease.

S. No.	Protein	Inhibitors	ES interactions	vdW interactions	Covalent bonds	Binding affinity (kcal/mol)
**1.**	Mprotease	N3	His163, His164, Phe140, Gly143, Glu166, His172, Gln189, Thr190, and Ala191	Thr26, His41, Thr24, Thr25, Met49, Ser144, Cys145, Leu141, Asn142, Asp187, Gln192, Met165, and Pro168	Cys145	–
**2.**	Mprotease	Psilacetin	His41, Tyr54, Glu166, Asp187 and Thr190	Met49, His164, Met165, Ley167, Pro168, Arg188, Gln198, Gln192	–	-6.0
**3.**	Mprotease	Psilocin	His41, Leu141, Cys145, His163, Gly143, Ser144, and Gln189	Thr25, Thr26, Leu27, Asn142, Met165, Phe140, Glu166, and His172	–	-5.4
**4.**	Mprotease	Psilocybine	His41, Asn142, Phe140, Leu141, Gly143, His163, Ser144, Cys145, Met165, His164, and Gln189	Thr25, Thr26, Leu27, Met49, Glu166, and His172	–	-5.8
**5.**	HIK-6	NAG, BMA, MAN, NDG	Asn110, Glu144, Gln147, Gln158, and Asn226,	Ser109, Val112, Pro145, and Ser227	Asn226	–
**6.**	HIK-6	Psilacetin	Ser106, Asn110, Gln158, Ser224, and Asn226	Phe103, Lys105, Ser109, and Val112	–	-4.1

### Interactions With Human Interleukin-6 Receptor

**Figure 2:**
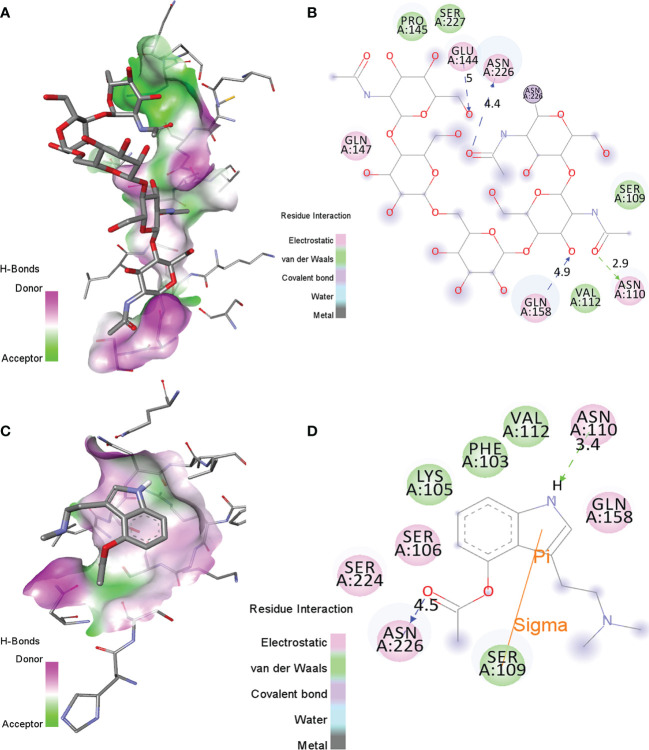
**(A, B)** The molecular interactions of the Human Interleukin-6 receptor with its co-crystallized ligand in the active site. **(C, D)** The molecular contacts of psilacetin with HIK-6.

The binding orientations of the co-crystallized ligand with human interleukin-6 receptor (PDB: 1N26) were analyzed. The co-crystallized ligand showed electrostatic interactions with residues Asn110, Glu144, Gln147, Gln158, and Asn226 of HIK-6. Further, it shows van der Waals interactions with residues Ser109, Val112, Pro145, and Ser227 of HIK-6. Additionally, it forms one covalent bond with residue Asn226 (**Figures 2A, B**). The binding orientations of psilacetin with HIK-6 were analyzed by superimposing with the co-crystallized ligand present in the active pocket of HIK-6. The binding energy of psilacetin with HIK-6 was found to be -4.1 kcal/mol. The psilacetin showed electrostatic interactions with residues Ser106, Asn110, Gln158, Ser224, and Asn226 of HIK-6. Further, it shows van der Waals interactions with residues Phe103, Lys105, Ser109, and Val112 of HIK-6 (**Figure 2C, D**).

SARS-CoV can be transmitted through air beads to the lungs. At this point, the infection enters the bloodstream which decreases CD4+, CD8+ T cells, and increases Th17 which is supportive of proliferative cells. As a result, pro-inflammatory cytokines are released, which contribute to the cytokine storm in COVID-19 pathogenesis. The cytokine storm is caused by rapid increases in pro-inflammatory cytokines such as IL-1, IL-6, and IL-8. The provocative cytokines are viewed as one of the major contributing components for serious COVID-19. Several analyses revealed that the cytokine storm is involved in multi-organ failure, destabilization of endothelial cell-to-cell collaborations, lung injury, and eventually death.

A severe inflammatory response and cytokine storm are mediated by IL-6. It is reported that IL-6 is elevated in COVID-19, especially in severely ill patients (41). In another study, increased levels of IL-6 in the blood was associated with a fatal outcome in COVID-19 infections. (42). The early detection of cytokine storms and fast treatment can help to defeat COVID-19 symptoms. This study shows that molecular interactions of psilacetin with the active pocket of HIK-6, and thereby blocking IL-6 could be possibly favorable for patients with severe lung inflammation caused by a cytokine storm. psilacetin binds to a membrane-bound IL-6 receptor, hence stopping IL-6 signaling and inflammatory response.

In contrast, the S glycoprotein on the envelope of the CoV binds to the ACE2 receptor on the membrane of host cells, which allows the virus to go into the cells. After binding, the coronavirus is activated, which results in the discharge of viral-RNA into the cytoplasm that leads to infection. Intact ACE2 is integrated with the virus in coronavirus infection. Several researches indicated that SARS-CoV infection can down-regulate the expression of ACE2 resulting in a physiological imbalance between angiotensin and ACE2 that could cause multiple organ injuries. It is reported that a few proteins and proteinases are perhaps engaged in the binding and membrane fusion activity (43). The ability of SARS-CoV2 to replicate in host cells depends on its Mprotease. The Mprotease of SARS-CoV-2 is an important enzyme that is crucial in conciliating viral transcription and replication by processing the polyproteins (44). The SARS-CoV-2 Mprotease cleaves the overlaying pp1a and pp1ab polyproteins to functional proteins that is a crucial level in viral replication. An attractive drug that targets to block Mprotease of SARS-CoV-2 could aid in inhibiting the processing of polyproteins. The present study shows the molecular interactions of psilacetin, psilocin, and psilocybine with the active pocket of SARS-CoV-2 Mprotease and could be resulted in stopping the RNA replication and thus lessen the symptoms of coronavirus.

### Structural Dynamics

**Figure 3:**
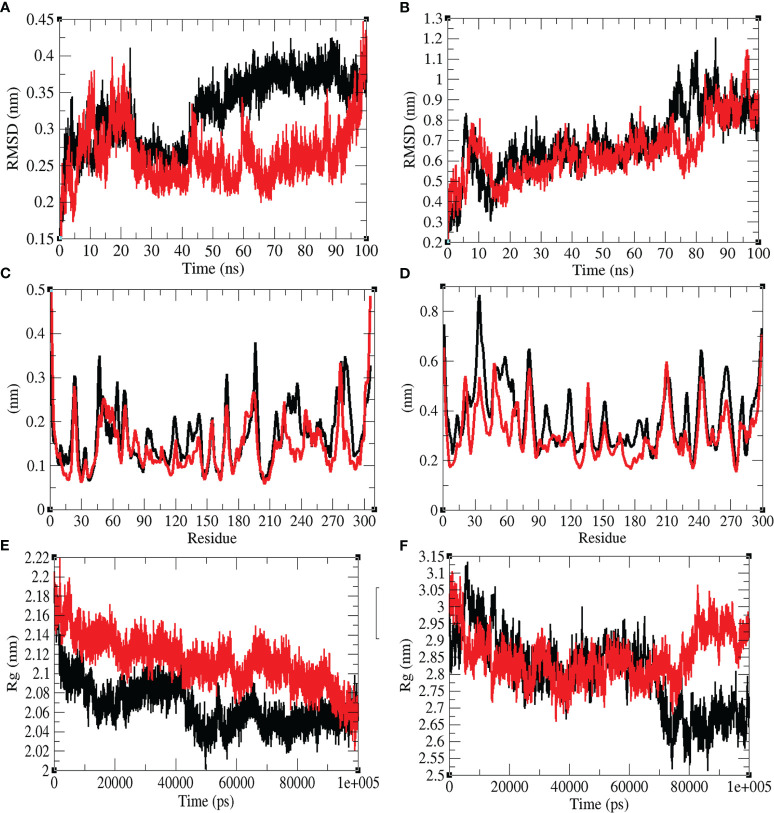
Structural dynamics. **(A)** RMSD plot for Mprotease (black) and Mprotease-psilacetin (red) vs. time. **(B)** RMSD plot for HIK6 (black) and HIK6- psilacetin (red) vs. time. **(C)** RMSF vs. residues for Mprotease (black) and Mprotease-psilacetin (red). **(D)** RMSF fluctuations vs. residues for HIK6 (black) and HIK6- psilacetin (red). **(E)** Radius of gyration (Rg) plot for Mprotease (black) and Mprotease-psilacetin (red). **(F)** Radius of gyration (Rg) plot for HIK6 (black) and HIK6-psilacetin (red).

The structural dynamics of Mprotease, Mprotease-psilacetin, HIK6, and HIK6-psilacetin were explored using the RMSD, RMSF, and the *R_g_*. The mean RMSD of Mprotease and Mprotease-psilacetin were 0.32 nm and 0.26 nm, respectively. The mean RMSD of HIK6, and HIK6-psilacetin were 0.69 nm and 0.65 nm, respectively. It has been discovered that RMSD values and residual fluctuations decrease upon binding of psilacetin to Mprotease and HIK6 (**Figure 3**). The mean *R_g_* for Mprotease and Mprotese-psilacetin were 2.07 nm and 2.11 nm, respectively. The mean *R_g_* for HIK6, and HIK6-psilacetin were 2.80 nm and 2.85 nm, respectively. It was discovered that Mprotease and HIK6 have less *Rg* and less compact packing due to attachment of psilacetin. The structural dynamics results indicate that attachment of psilacetin with Mprotease and HIK6 alter the structure dynamics and properties of these proteins.

**Figure 4:**
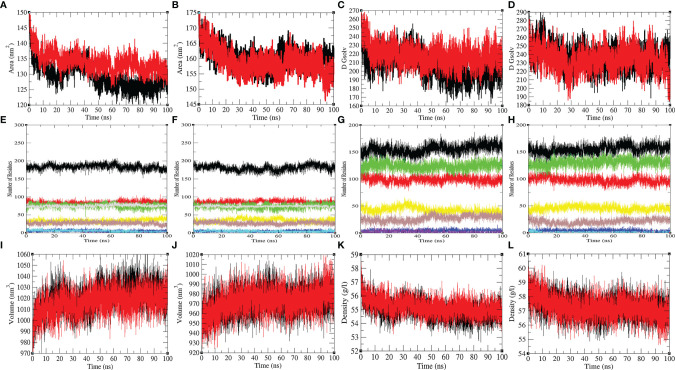
The SASA and secondary structure plot. The SASA plot of **(A)** Mprotease (black) and Mprotease-psilacetin (red), **(B)** HIK6 (black) and HIK6-psilacetin (red). The free energy of solvation for **(C)** Mprotease (black) and Mprotease-psilacetin (red), **(D)** HIK6 (black) and HIK6-psilacetin (red). The graphical representation indicates structural elements present in **(E)** Mprotease, **(F)** Mprotease-psilacetin, **(G)** HIK6, and **(H)** HIK6-psilacetin. The structural volume **(I)** Mprotease (black) and Mprotease-psilacetin (red), **(J)** HIK6 (black) and HIK6-psilacetin (red), and the density of **(K)** Mprotease (black) and Mprotease-psilacetin (red), **(L)** HIK6 (black) and HIK6-psilacetin (red) calculated during MD simulations.

The mean SASA for Mprotease and Mprotease-psilacetin were found to be 129.55 nm^2^ and 134.01 nm^2^, respectively. The mean SASA for HIK6 and HIK6-psilacetin were 160.23 nm^2^ and 159.05 nm^2^, respectively (**Figure 4**). The SASA scheme indicated that core amino acids in Mprotease are uncovered to solvent molecules after psilacetin attaches to it. There SASA are not affected much for HIK6. Further, the solvation energy of Mprotease, Mprotease-psilacetin, HIK6, and HIK6-psilacetin were found to be 207.35, 218.54, 237.03, and 233.75 kJ/mol/nm^2^, respectively. The solvation energy of Mprotease-psilacetin was more than Mprotease, while HIK6-psilacetin has less solvation energy than HIK6.

Further, the secondary structure allocations in Mprotease, Mprotease-psilacetin, HIK6, and HIK6-psilacetin were highlighted at each period. The mean amino acid residues involved in structure development of Mprotease, Mprotease-psilacetin, HIK6, and HIK6-psilacetin were noted (**Table 3**). It was found that the mean amino acid contributed in structure formation for Mprotease, Mprotease-psilacetin, HIK6, and HIK6-psilacetin were 60%, 59%, 52%, and 52%, respectively (**Figures 3C, D**). It has been found that binding of psilacetin inhibits and unfolds β-sheet and α-helix of Mprotease. In the case of HIK6, there were no such changes in the secondary structure reported. The volume of Mprotease, Mprotease-psilacetin, HIK6, and HIK6-psilacetin were 54.98 nm^3^, 55.23 nm^3^, 57.30 nm^3^, and 57.27 nm^3^, respectively. The average density of Mprotease, Mprotease-psilacetin, HIK6, and HIK6-psilacetin were found to be 1020.79 g/l, 1016.11 g/l, 971.59 g/l, and 972.14 g/l, respectively. There were slight changes reported when psilacetin binds to Mprotease and HIK6.

**Table 3:** Percentage of amino acid residues present in Mprotease, Mprotease-psilacetin, HIK6, and HIK6-psilacetin during MD simulations.

Protein	Secondary structure (%)							
	Structure*	Coil	β-sheet	β-bridge	Bend	Turn	α-helix	3_10_-helix
Mprotease	60	27	25	2	11	9	25	2
Mprotease-psilacetin	59	28	24	2	11	9	24	2
HIK6	52	33	42	1	14	9	0	1
HIK6-psilacetin	52	33	44	1	15	7	0	0

*Structure = α-helix + β-sheet + β-bridge + Turn.

### PCA and GFE Landscape

The PCA shows a general expansion of Mprotease, Mprotease-psilacetin, HIK6, and HIK6-psilacetin during the course of simulation. It classifies mean fluctuations and atomic mobility of Mprotease, Mprotease-psilacetin, HIK6, and HIK6-psilacetin. The mean of eigenvalues is an amount of the total motion in the system. It also estimates the flexibility of a molecule. The eigenvalues were found to be 383.19 nm^2^, 295.54 nm^2^, 1825.15 nm^2^, and 1247.63 nm^2^, for Mprotease, Mprotease-psilacetin, HIK6, and HIK6-psilacetin, respectively. It was calculated to be more for Mprotease and HIK6 than Mprotease-psilacetin and HIK6-psilacetin, respectively. Lesser values in case of Mprotease-psilacetin and HIK6-psilacetin may represent strong attachment of psilacetin.

**Figure 5:**
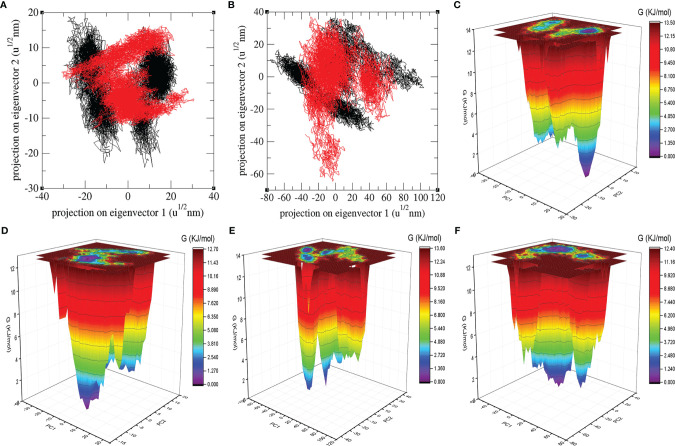
Atomic projections and Gibbs energy landscape. The atomic projections on eigenvectors indicated different states of **(A)** Mprotease (black) and Mprotease-psilacetin (red), **(B)** HIK6 (black) and HIK6-psilacetin (red). The GFE landscapes are plotted for **(C)** Mprotease, **(D)** Mprotease-psilacetin, **(E)** HIK6, and **(F)** HIK6-psilacetin, respectively.

The colourful GFE landscape showed diverse forms for Mprotease, Mprotease-psilacetin, HIK6, and HIK6-psilacetin, respectively (**Figure 5**). The GFE landscape with deep blue colour indicates lower energy state. Other blue regions describe changes in the molecular conformation of Mprotease, Mprotease-psilacetin, HIK6, and HIK6-psilacetin. It was found that the lowest energy regions of Mprotease, Mprotease-psilacetin, HIK6, and HIK6-psilacetin are dissimilar in each event. A pictorial assessment among the GFE values of Mprotease, Mprotease-psilacetin, HIK6, and HIK6-psilacetin, indicated different outlines of global minima. The native state of Mprotease and HIK6 showed sharp global minima, while binding of psilacetin changes the pattern. This might be due to inhibition of proteins by psilacetin.

## Conclusion

On 30 Jan (2020), the WHO declared COVID-19 pandemic as a Public Health Emergency of International Concern. The current investigation suggested that the psilocybin-mushroom could be utilized for the treatment of COVID-19 infections. Our hypothesis and computational calculations suggested that the psychedelic compounds could bind and inhibit Mprotease of SARS-CoV-2 and HIK-6. The strong binding of psilacetin leads to decrease in atomic and residual fluctuations of Mprotease and HIK-6. The binding of psilacetin shows different eigenvalues and Gibbs free energy pattern in Mprotease and HIK-6. Even though detailed binding analysis of psilacetin to Mprotease and HIK-6 were performed, it has several possible research limitations. Further experiments are required to back these outcomes.

## Data Availability Statement

The original contributions presented in the study are included in the article/supplementary materials, further inquiries can be directed to the corresponding author.

## Author Contributions

Conceptualization, FK and DL. Methodology, FK. Software, FK. Validation, FK and DL. Formal analysis, FK., FH., and DL. Investigation, FK. and DL. Resources, FK and DL. Data curation. FK and DL. Writing—original draft preparation, FK, FH, and DL. Writing—review and editing, FK. Visualization, FK. Supervision, DL. Project administration, DL. Funding acquisition, FK and DL. All authors have read and agreed to the published version of the manuscript.

## Funding

The Sichuan Science and Technology Program (2021YFH0093), and the China Postdoctoral Science Foundation (2020M 673187) supported this work.

## Conflict of Interest

The authors declare that the research was conducted in the absence of any commercial or financial relationships that could be construed as a potential conflict of interest.

## Publisher’s Note

All claims expressed in this article are solely those of the authors and do not necessarily represent those of their affiliated organizations, or those of the publisher, the editors and the reviewers. Any product that may be evaluated in this article, or claim that may be made by its manufacturer, is not guaranteed or endorsed by the publisher.
